# Efficiently Predicting Vancomycin Resistance of *Enterococcus Faecium* From MALDI-TOF MS Spectra Using a Deep Learning-Based Approach

**DOI:** 10.3389/fmicb.2022.821233

**Published:** 2022-06-06

**Authors:** Hsin-Yao Wang, Tsung-Ting Hsieh, Chia-Ru Chung, Hung-Ching Chang, Jorng-Tzong Horng, Jang-Jih Lu, Jia-Hsin Huang

**Affiliations:** ^1^Department of Laboratory Medicine, Chang Gung Memorial Hospital, Taoyuan, Taiwan; ^2^Ph.D. Program in Biomedical Engineering, Chang Gung University, Taoyuan, Taiwan; ^3^Taiwan AI Labs, Taipei, Taiwan; ^4^Department of Computer Science and Information Engineering, National Central University, Taoyuan, Taiwan; ^5^Department of Bioinformatics and Medical Engineering, Asia University, Taichung, Taiwan; ^6^School of Medicine, Chang Gung University, Taoyuan, Taiwan; ^7^Department of Medical Biotechnology and Laboratory Science, Chang Gung University, Taoyuan, Taiwan

**Keywords:** vancomycin-resistant *Enterococcus faecium* (VRE*fm*), antibacterial drug resistance, MALDI-TOF MS, convolutional neural network (CNN), rapid detection

## Abstract

Matrix-assisted laser desorption ionization time-of-flight (MALDI-TOF) mass spectrometry (MS) has recently become a useful analytical approach for microbial identification. The presence and absence of specific peaks on MS spectra are commonly used to identify the bacterial species and predict antibiotic-resistant strains. However, the conventional approach using few single peaks would result in insufficient prediction power without using complete information of whole MS spectra. In the past few years, machine learning algorithms have been successfully applied to analyze the MALDI-TOF MS peaks pattern for rapid strain typing. In this study, we developed a convolutional neural network (CNN) method to deal with the complete information of MALDI-TOF MS spectra for detecting *Enterococcus faecium*, which is one of the leading pathogens in the world. We developed a CNN model to rapidly and accurately predict vancomycin-resistant *Enterococcus faecium* (VRE*fm*) samples from the whole mass spectra profiles of clinical samples. The CNN models demonstrated good classification performances with the average area under the receiver operating characteristic curve (AUROC) of 0.887 when using external validation data independently. Additionally, we employed the score-class activation mapping (CAM) method to identify the important features of our CNN models and found some discriminative signals that can substantially contribute to detecting the ion of resistance. This study not only utilized the complete information of MALTI-TOF MS data directly but also provided a practical means for rapid detection of VRE*fm* using a deep learning algorithm.

## Introduction

Matrix-assisted laser desorption ionization time-of-flight (MALDI-TOF) mass spectrometry (MS) has become a promising analytical technique in many clinical microbiology laboratories in identifying bacterial species. However, applying MALDI-TOF MS in determining antibiotic susceptibility test (AST) has not been widely developed. The traditional approach utilized the presence or absence of several peaks on MS spectra to predict the AST (Wolters et al., 2011; Lasch et al., 2014). However, the predictive performance based on the traditional approach is discrepant from each other, hindering the application in clinical settings. The discrepancy in predictive performance would have resulted from the insufficient number of peaks used in these studies. Moreover, single peaks' presence or absence rather than the whole pattern of the peaks were used for classifying AST. In the past few years, some studies have harnessed artificial intelligence (AI) algorithms to analyze the MALDI-TOF MS peaks pattern for classifying specific bacterial strains (Wang et al., 2019, 2020b; Weis et al., 2022). Most of the works studied *Staphylococcus aureus* (Weis et al., 2022), group B *Streptococcus* (Wang et al., 2019), and Enterobacteriaceae (Weis et al., 2022). By contrast, *Enterococcus faecium* is also a superbug with rising clinical importance (Ahmed and Baptiste, 2018), only a few studies have been reported for rapid detection of vancomycin-resistant *Enterococcus faecium* (VRE*fm*) by using MALDI-TOF MS and machine learning approaches (Griffin et al., 2012; Wang et al., 2021). Rapid detection of VRE*fm* would result in favorable clinical outcomes, including reduced mortality rate and reduced use of broad-spectrum antibiotics for severe VRE*fm* infection.

A recent benchmark study has demonstrated that a one-dimensional convolutional neural network (CNN) outperformed traditional machine learning methods for the bacterial species identification based on the MALDI-TOF MS data (Mortier et al., 2021). Deep learning methods, such as CNNs and recurrent neural networks (RNNs), have been repeatedly proved to outperform the classical machine-learning algorithms for large datasets with high-dimensional data. A CNN applies filters in the form of a convolution operation to extract features from the data. The advantage of a CNN is that it reduces the parameters compared to other neural networks by sharing them as multiple filters (Lecun et al., 1998). The convolution method, which is coupled with a small grid of input signals into local receptive features, could be a solution to solve the peak shift among samples to apply raw data directly. Furthermore, the convolution filters share the parameters independent of position; thus, we can reduce the number of used parameters. This parameter sharing of the convolutional filters and the local connections of the nodes increases the performance in handling sparsely connected data.

In this study, we aim to propose a CNN algorithm for practical extraction and analysis of multidimensional MS spectral data for VRE*fm* prediction. By using the consecutively collected MALDI-TOF MS data from large clinical isolates from two tertiary medical centers (Chang Gung Memorial Hospital [CGMH], Linkou branch, and Kaohsiung branch) in Taiwan (Wang et al., 2021), we coupled with the respective laboratory-confirmed antibiotic resistance profile to build CNN models for antimicrobial resistance prediction. We first demonstrated the efficacy of our CNN architectures for discrimination of VRE*fm* from vancomycin-susceptible *Enterococcus faecium* (VSE*fm*) isolates in reporting the area under the receiver operating characteristic (AUROC) value over 0.800. Next, a full CNN model trained with data from the Linkou branch achieved good performance with an average AUROC of 0.887 in predicting VRE*fm* in the independent data from the Kaohsiung branch. Finally, we applied the Score-CAM method and statistical analysis to examine the important features that the CNN models used to predict VRE*fm* isolates. Of note, some essential features of the CNN model were reported in the literature and many m/z ranges were novel features, showing significant differences in m/z intensities of the MALDI-TOF MS spectra in the VRE*fm* from clinical susceptible isolates. Furthermore, since mass spectra can be generated rapidly from colonies following an overnight culture, we provide an efficient CNN framework to build a prediction model for antimicrobial resistance based on the complete MALDI-TOF MS profiles directly and, therefore, could help the clinical management of patients with infectious diseases.

## Materials and Methods

### Data Sources

Matrix-assisted laser desorption ionization time-of-flight MS spectra of VRE*fm* were used as the input features, while the susceptibility test to vancomycin was used as the label of interest. MALDI-TOF MS (Bruker Daltonik GmbH, Bremen, Germany) spectra of VRE*fm* were consecutively collected between 2013 and 2017 in Chang Gung Memorial Hospital, Linkou, and Kaohsiung branches. The manufacturer's instruction was followed, and default settings were used to identify *E. faecium* (Wang et al., 2018a). Biotyper 3.1 software (Bruker, Germany) was used for species identification of *E. faecium*. Regarding labeling, the susceptibility to vancomycin was determined by using the paper disc method based on the CLSI M100 guideline (CLSI, 2020). Accordingly, the detailed specimen distribution of VRE*fm* and VSE*fm* clinical isolates is summarized in Supplementary Figures 1A,B in the Linkou and Kaohsiung branches, respectively. The MALDI-TOF spectra were preprocessed by using Flexanalysis (Bruker, Germany). The relative intensity threshold was set as zero. The signal-to-noise ratio (S/N ratio) of two was used to filter out signals whose S/N ratio was lower than two. Baseline subtraction of the raw spectra was done by using the top-hat algorithm. Savitzky-Golay algorithm was adopted to smooth the spectra. MS spectra preprocessed by the above methods were used as the input data for the subsequent modeling.

### CNN Model

In this study, we used one-dimensional CNNs to classify the *E. faecium* strain with vancomycin resistance (VRE*fm*) according to the raw MS data. Herein, the MS raw data represent the input data and the goal is to predict whether the given sample is VRE*fm* (class label 1) or not (VSE*fm*, class label 0). In total, 7997 *E. faecium* cases were identified and included from the Linkou and Kaohsiung branches of CGMH, while 4,017 cases were VRE*fm* (50.23%) and 3,980 cases were VSE*fm* (49.77%) cases, respectively.

**Figure 1:**
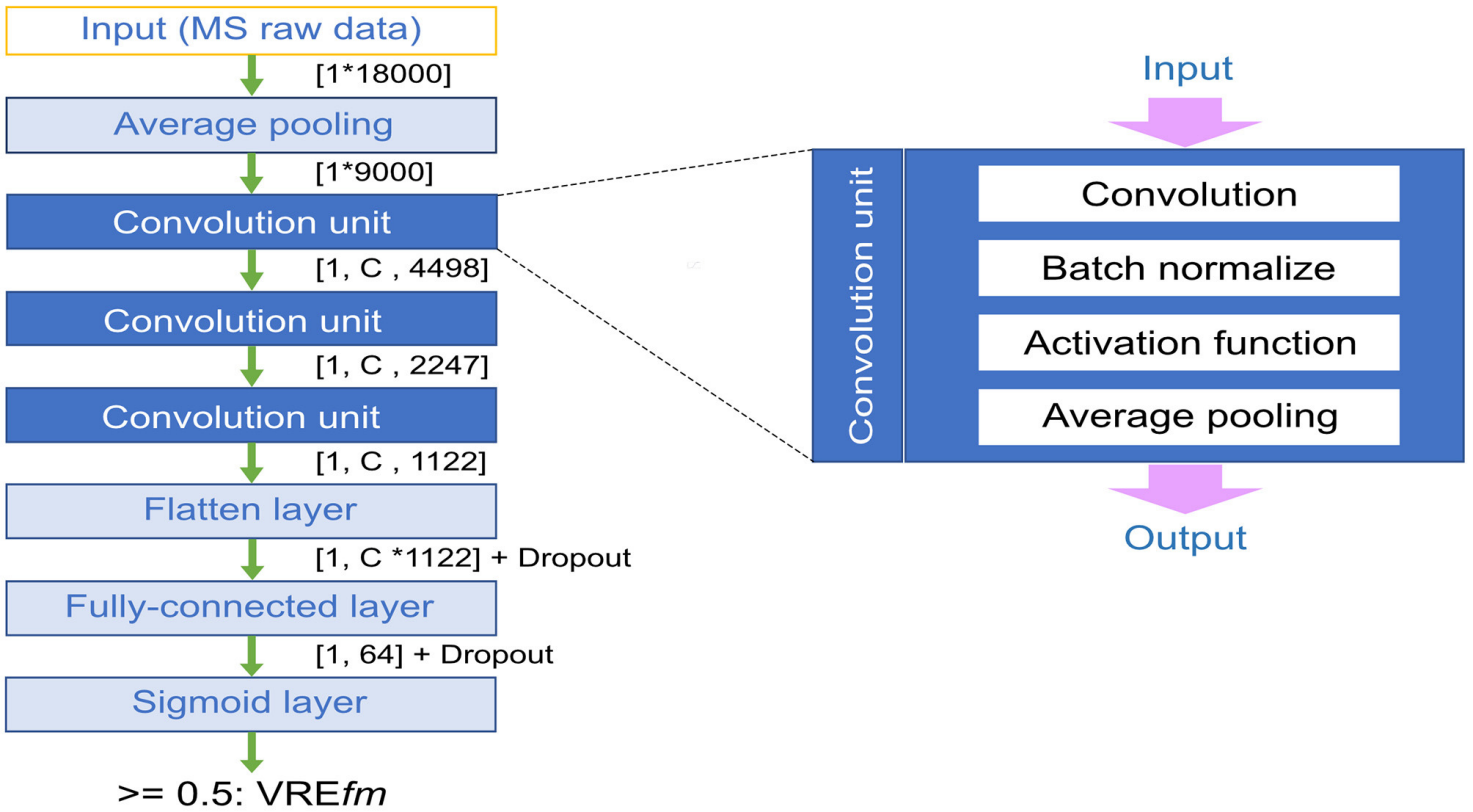
Illustration of convolutional neural network (CNN) model architecture. C denotes different channels, and the dropout rate was 0.5.

Figure 1 shows a CNN model architecture used in this study. Because the whole m/z signals of raw MS data represented a vector consisting of 18,000 values, an average pooling (avg pooling) layer is used for the summation of raw signals from the nearby m/z peaks, which are sometimes shifted among samples. A CNN model can learn local m/z signal patterns, which are good discriminators between positive and negative instances in the training dataset. We added three convolutional layers to learn the information across the patterns of m/z peaks. The information of adjacent m/z signals is embedded in the entries of kernels (filters) used in the first convolution layer. Additional CNN layers can learn higher-order interactions between signals at different m/z regions. A convolution layer comprises four units, including convolution filter or kernel, batch normalization, activation function, and pooling. After the convolution, a flattened layer was applied to conjugate the high-level information of m/z peak patterns.

In this study, we tried several versions of CNN models including preserving the average pooling layer (on or off), different activation functions using tanh or rectified linear units (ReLu), and a varying number of channels in the convolution layers (32, 64, 128), and the dropout rate (0 or 0.5) in the final fully connected layer. The output layer contained a single neuron with the Sigmoid activation function, which learns the mapping from the hidden (fully connected) layer to the output class labels [0, 1]. The final output is a probability indicating whether an input is a vancomycin-resistant *E. faecium* strain.

### Training and Testing

To develop a robust CNN model, we applied 10-fold cross-validation in the data from the CGMH Linkou branch to evaluate the model performance for the optimal CNN architecture. Next, the whole data from the CGMH Linkou branch were used as the training set to develop the final CNN model; the data from the CGMH Kaohsiung branch served as the unseen independent testing set for the external validation. In making a performance comparison on the testing set, we also applied two commonly used machine learning (ML) algorithms, i.e., random forest (RF) (Breiman, 2001) and extreme gradient boosting (XGBoost) (Chen and Guestrin, 2016), to build the VRE*fm* prediction models. For the ML-based models, the input attributes of the important peaks were selected by the feature selection method used in the previous study (Wang et al., 2021). The primary parameters of the two ML models were summarized in Supplementary Table 1. In addition, we developed those models using ten different random seeds for performance evaluation and feature selection.

### Performance Measurement of Predictive Models

The VRE*fm* prediction models trained using CNN algorithms were evaluated *via* 10-fold cross-validation (CV) using the data from the Linkou branches of CGMH. In the 10-fold CV, all the data from Linkou branches of CGMH were randomly divided into ten subgroups with approximately equal data sizes. Each fold of subgroups was used as the independent testing data to evaluate the performance of the model trained on the other dataset. To evaluate the model performance, accuracy (ACC) and the area under the receiver operating characteristic (AUROC) were considered as the primary metrics for the performance comparison. After obtaining the CNN model architecture with better performance, all data from the Linkou branches of CGMH were used to train a full model. The data from the Kaohsiung branch of CGMH served as the unseen independent testing data for external validation, method comparison, and feature selection.

### Feature Selection Procedures

We selected the top and last 500 samples from the CGMH Kaohsiung branch data according to the prediction scores from the best CNN models. In order to see where the CNN model learned from the MS peak patterns, we applied the selected samples to verify the important weight of each m/z peak of the CNN models using the Score-CAM technique (Wang et al., 2020a). First, we ranked the Score-CAM scores of each m/z peak, chose the highest 1% of peaks and conjugated the adjacent m/z peaks into a range. Then, the informative features were identified if the m/z peak ranges were selected at least eight times among ten independent CNN models.

We further investigated the peak intensities of the informative features and box plots were adopted to display their distributions. For each m/z peak, the intensity values of all samples from the CGMH Kaohsiung branch were normalized by the *Z*-score transformation. Then, the normalized *Z*-scores of the given m/z peaks within the informative features were averaged for every sample. Of note, for a given informative feature, a few isolates were removed from statistical comparison when the intensity for the feature was zero.

### Statistics

Statistical analysis was performed by using R software version 4. The differences of multiple groups were analyzed by one-way ANOVA and followed by the Tukey *post hoc* test. In addition, the differences in the normalized intensity of the informative features between VRE*fm* and VSE*fm* isolates were calculated by Wilcoxon rank-sum test and the significant level was controlled for multiple testing using the *q-value* method (Storey et al., 2020).

### Code Availability

The computer codes that support the findings of this study were deposited to the GitHub repository and are available at https://github.com/p568912/CNN_MALDI-TOF_VREfm.

## Results

### Development of Deep Learning-Based VRE*fm* Prediction Models

We aimed to develop a robust deep learning-based VRE*fm* prediction model using the complex MALDI-TOF m/z spectra without predictive peak selection in the data preprocessing as shown in the previous study (Wang et al., 2021). We used the cases from the CGMH Linkou branch to evaluate the architectures and parameters of the CNN models with a 10-fold cross-validation approach. Next, we compared CNN model performance with the other two machine learning algorithms using the cases from the CGMH Kaohsiung branch as the unseen independent testing dataset. Finally, we trained ten CNN models independently by setting different seeds for model initiation and examining the prediction performance with two matrices including ACC and AUROC.

**Figure 2:**
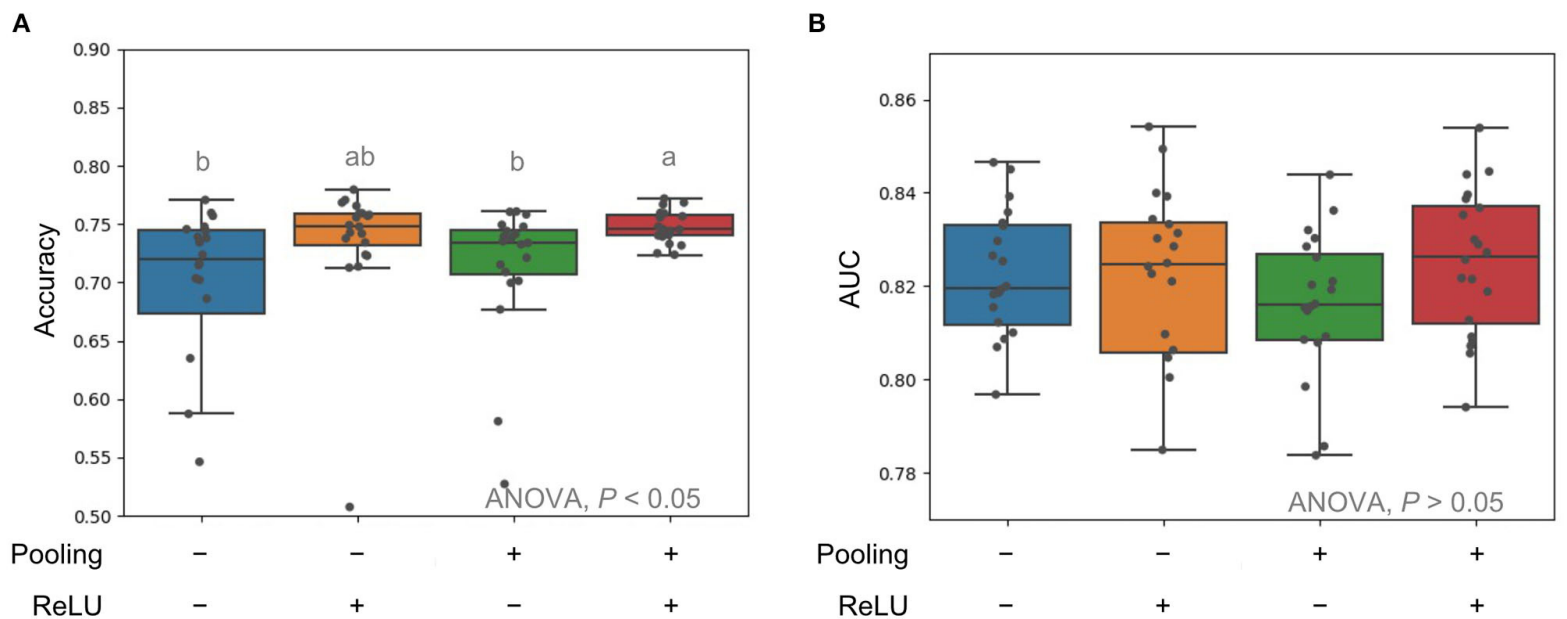
Comparison of average pooling in terms of accuracy **(A)** and AUC **(B)** for input mass spectrometry (MS) raw data and rectified linear units (ReLu) as activation function. Multiple comparison tests on the different units were applied in the models using one-way ANOVA, following the *post-hoc* Tukey honest significant difference (HSD) test for paired difference with a *p*-value < 0.05. Different letters indicate a significant difference.

We first tested different CNN architectures with the pooling techniques on the input data and different activation functions. As the pooling layer provides an approach to summarize the MS peak intensities across the nearby region, the slight shift of MS peaks across samples is expected to be solved. Indeed, the variation of accuracies across 10-fold CV was much reduced when the presence of a pooling layer (Figure 2A). When ReLu as the non-linear activation function was used in the neurons, the model performance with a prior pooling layer yielded the best performance in the models (Figure 2B).

**Figure 3:**
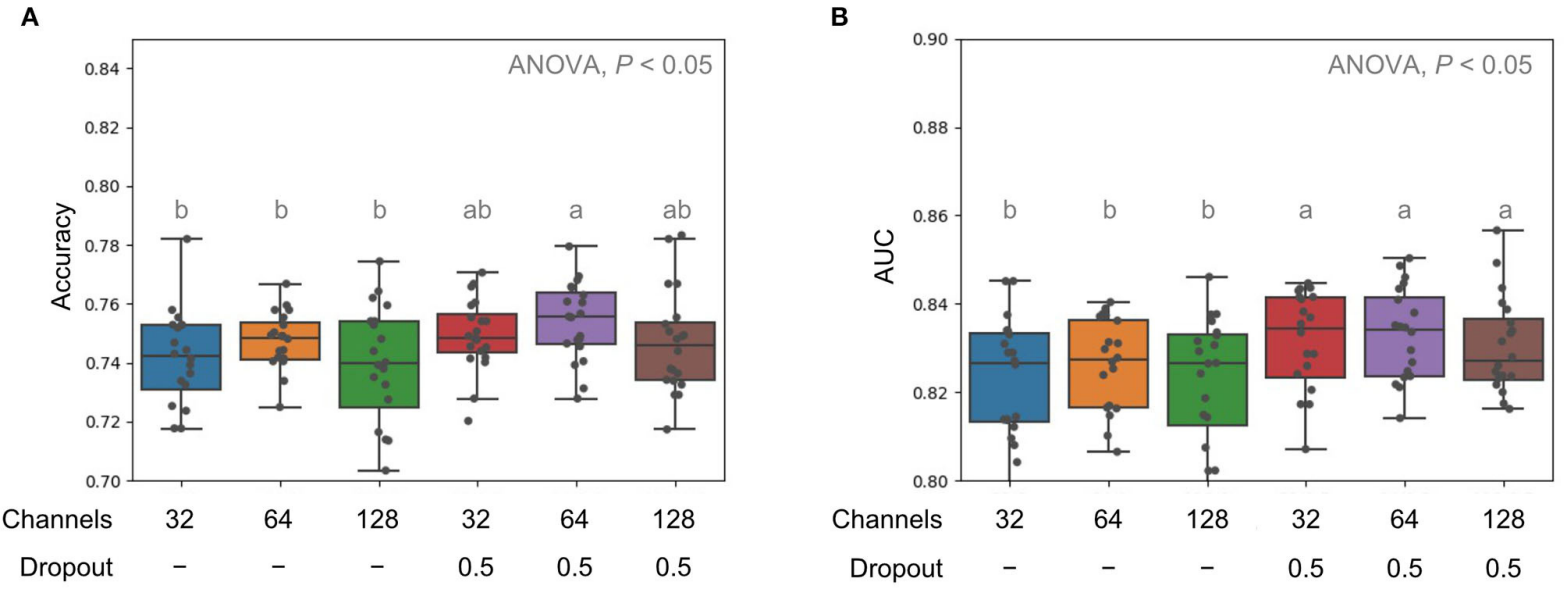
Comparison of model performance in terms of accuracy **(A)** and AUC **(B)** for with different channel sizes and dropouts. Multiple comparison tests on the different units were applied in the models using one-way ANOVA, following the *post-hoc* Tukey HSD test for paired difference with a *p*-value < 0.05. Different letters indicate a significant difference.

Next, we tried to decrease or increase the channel sizes to 32 and 128, respectively, for each convolution layer and added dropout, which could avoid the overfitting problem. Without dropout function, the ACC and AUROC of those models with lower (32) or higher (128) channels were generally lower than the models with a channel size of 64 (Figure 3A). However, we observed that the proceeding dropout during training could significantly improve the performance in prediction by showing better AUROC values (Figure 3B).

### Comparison Between Machine Learning Models and CNN Models

**Figure 4:**
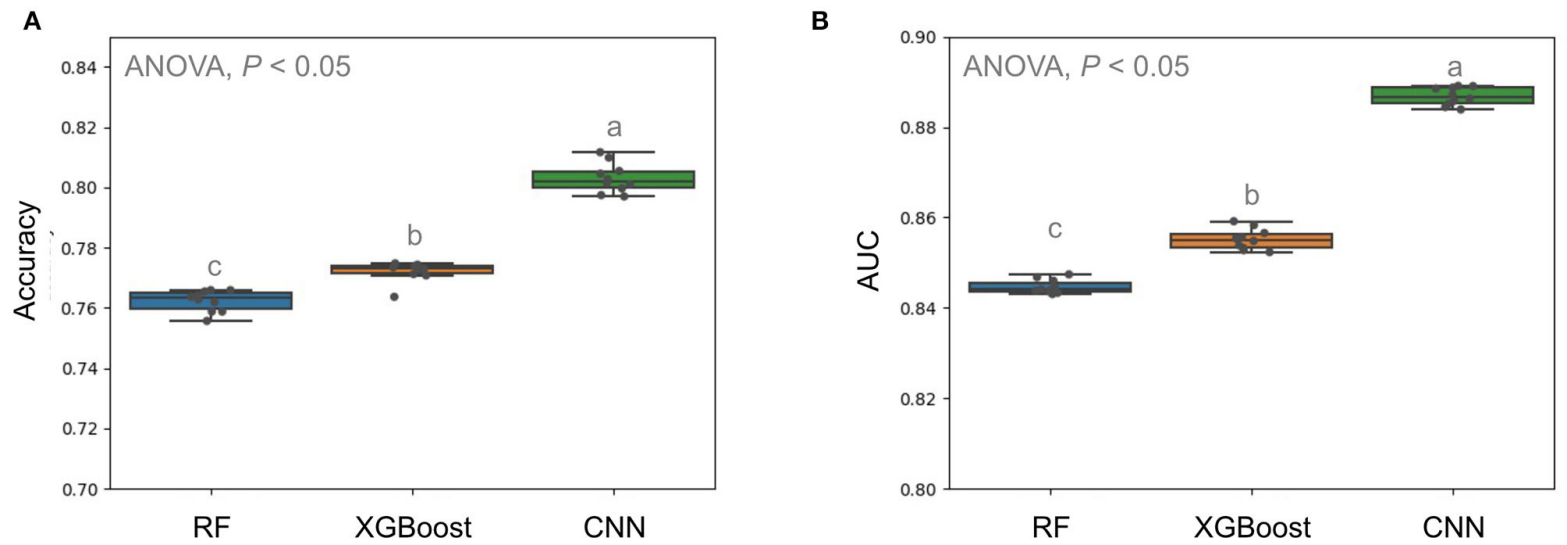
Comparison of prediction performance in terms of accuracy **(A)** and AUC **(B)** using different models. Multiple comparison tests on the different units were applied in the models using one-way ANOVA, following the *post-hoc* Tukey HSD test for paired difference with a *p*-value < 0.05. Different letters indicate a significant difference.

The previous study applied a decision tree-based algorithm named RF to predict the VRE*fm* based on the selected m/z peaks from manual alignment and statistical tests (Wang et al., 2021). Accordingly, we constructed RF-based classifiers and XGBoost-based classifiers with the selected m/z peaks and compared them to our CNN models using whole MS spectra. In this study, the MALDI-TOF MS spectra obtained from the CGMH Kaohsiung branch were regarded as the independent testing dataset. To evaluate the performance fairly, the training dataset was from the Linkou branch and the cases from the Kaohsiung branch was set as unseen testing dataset. The results showed in Figure 4. CNN models attained higher performance in predicting VRE*fm* (average accuracy = 0.796) than RF and XGBoost (average ACC = 0.762 and 0.772, respectively). Furthermore, the average AUROC of the CNN algorithm achieved a better performance of 0.887 than the other RF and XGBoost algorithms (average AUROC = 0.845 and 0.855, respectively) (Figure 4B).

### CNN Models Capture Important Features to Predict VRE*fm*

**Figure 5:**
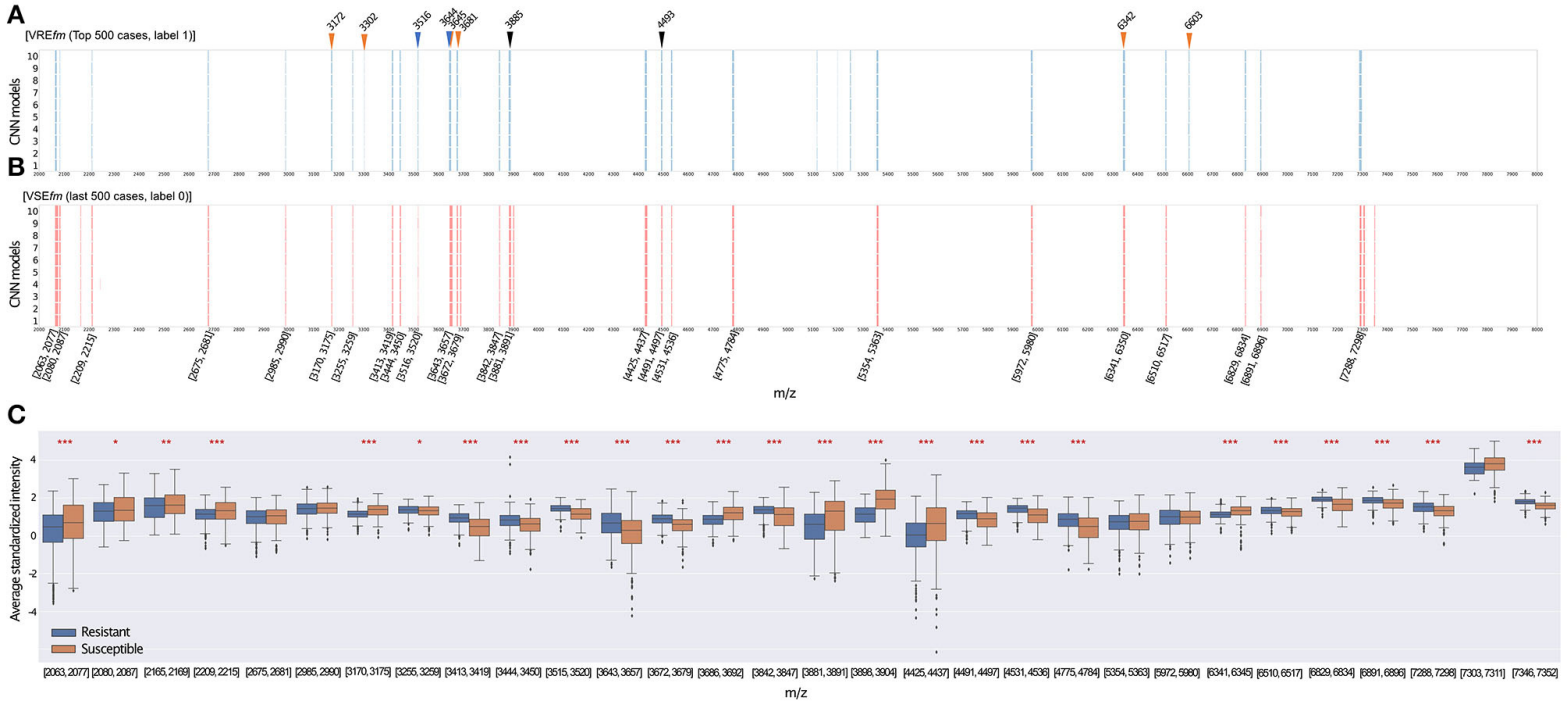
Feature selection is based on the Score-CAM. Each row of m/z peaks represents the important features with top 1% weights in the different models. The important features of our CNN models for the classification of vancomycin-resistant *E. faecium* (VRE*fm*) **(A)** and vancomycin-susceptible *E. faecium* (VSE*fm*) **(B)** in the last and top 500 testing cases, respectively. Rectangles represent the important features of m/z ranges selected by at least 8 independent CNN models using Score-CAM. Triangles indicate that the important markers at m/z signals for discrimination of VRE*fm* strain have been identified in the literature. Blue is identified by Wei et al. (2014), black is identified by Lasch et al. (2014), and orange is identified by Wang et al. (2020b). **(C)** The intensity distribution of informative features with the normalized intensities of 500 resistant and 500 susceptible isolates. The star (*) indicates a statistical difference in the feature intensities between resistant and susceptible isolates. Wilcoxon-rank sum test was applied to test the difference between the two groups. * *q*-value < 0.05, ** *q*-value < 0.01, ***, *q-*value < 0.001.

We applied the Score-CAM (Wang et al., 2020a) to examine the importance of m/z signals as informative features to affect the prediction performance of the best model for the top 500 VRE*fm* and VSE*fm* prediction scores, respectively. Score-CAM was introduced to visually explain how the CNN models classified the MALDI-TOF MS signal patterns into two groups. According to the Score-CAM scores and selection procedures (see Materials and Methods), the informative features as the predictive m/z peak ranges for making the final categorization decision as VRE*fm* or VSE*fm* were shown in Figures 5A,B, respectively. First, the informative features for classifying respective VRE*fm* and VSE*fm* were mostly overlapped. That is, the CNN models could evaluate the patterns of the mass peak ranges to distinguish VRE*fm* and VSE*fm* consistently. Second, many m/z peak signatures, have been reported to identify the vancomycin-resistant *E. faecium*. in the previous works of literature (Lasch et al., 2014; Wei et al., 2014; Wang et al., 2021), were included in the ranges of our informative features. For example, six out of ten most critical predictive peaks for VRE*fm* prediction in our previous work (Wang et al., 2021) have been identified as the important feature ranges of the CNN models. Third, several informative features were specific to classify VRE*fm* such as 3,301–3,304 Da, 5,114–5,118 Da, 5,197–5,200 Da, 5,247–5,253 Da, and 6,602–6,608 Da. In comparison with the statistical method to extract crucial peaks in our previous study (Wang et al., 2021), the occurrence of two peaks of m/z 3,302 and m/z 6,603 were concordantly found in the current CNN model.

We selected the 30 informative features based on the Score-CAM results and further illustrated the differences in intensities of spectra between VRE*fm* and VSE*fm* (Figure 5C). Notably, 25 out of 30 informative features in terms of their average m/z peak intensities showed a significant difference between the resistant and susceptible isolates (*q*-value < 0.05, Wilcoxon rank-sum test). The important features in the Score-CAM were apparently in accordance with the observed results of the MALDI-TOF signal profiles (Supplementary Figure 3). This investigation implied that the Score-CAM method successfully captured the critical signals to distinguish the VRE*fm* and VSE*fm* isolates by the CNN model. More importantly, the results provide an explanation that our CNN models could automatically detect the important features to obtain high performance reasonably.

## Discussion

This study focused on the classification of resistance strain typing of *E. faecium* based on the MALDI-TOF MS utilizing CNN algorithm method directly. Specifically, the complete raw spectra data did not need to undergo the time-consuming preprocessing steps such as peak alignment of individual samples and statistical analysis to filter out crucial peaks. Instead, we utilized the average pooling method to solve the problem of peak shift across samples and the deep learning technique to learn the complex interaction between m/z peaks related to the resistant and susceptible *E. faecium* strain typing. The rapid identification of VRE*fm* strain types will facilitate the speed in the identification of suspicious infections and provide suitable treatment to patients for rapid infection control. Additionally, we further explore the discriminative features in the CNN model, which was considered a black box, and will allow the identification of each corresponding peptide with further biological experiments as the followings. Such findings should provide clinically valuable information pertaining to the different subtypes of *E. faecium* and other resistant bacterial strains.

In order to handle the issue of peak shifting, alternative approaches were used including “type templates” for each susceptible type based on the incidence of specific peaks in their MALDI-TOF MS spectra (Wang et al., 2018a,b), recursive search based on the statistical analysis and performance of classifiers (Chung et al., 2019) and the embedded feature-selection method (Wang et al., 2021). Different approaches were designed to deal with the peak shift problem of using spectral data with particular procedures. In contrast, we here use the average pooling, which is a straightforward approach, to consider the peak shift problem to tolerant the difference across the spectra data. Although the average pooling might compromise the crucial peak values by the nearby unrelated peaks, the convolutional neural networks were applied to extract the important information among features with fully connected layers in each convolution unit (Figure 1). In addition, many peak values in the whole spectra data were zero. The introduction of the ReLu function overcame the vanishing gradient problem better than the sigmoid function as suggested for the image classification problems (Krizhevsky et al., 2017) in Figure 2. Therefore, the CNN architecture could be an easy and suitable solution for the classification of VRE*fm* and VSEf*m* isolates using whole MS spectra data.

Directly input of whole MS spectra data in the convolutional neural network could be suffered by the exploration of parameters and thus causes the overfitting problem in such networks. It has been demonstrated that dropout techniques are robust to prevent neural networks from overfitting (Hinton et al., 2012; Srivastava et al., 2014). We, therefore, applied the dropout in the later layers of CNN model architecture and yielded significantly better AUROC performance (Figure 3B). With a detailed examination of the CNN model architecture, the proposed CNN model in this study remarkably outperformed other ML models, which were developed in our previous works (Wang et al., 2021), for clinical application (Figure 4).

Although identification of crucial predictive peaks in VRE*fm* strains may not be essential in clinical application, interpreting CNN models is important to build people's confidence in the system and to facilitate future studies in the exploration of molecular mechanisms behind the resistance. The parameters of deep neural networks are usually large and difficult to be interpreted. However, several techniques such as saliency map (Hong et al., 2015) and Score-CAM (Wang et al., 2020a) have been developed to explain the crucial features in the deep neural networks successfully. In the feature selection section, most of the informative features have been reproducibly identified among 10 CNN models with randomly assigned initial values. That is, our proposed CNN models were robust enough to reach a globally optimal solution to predict VRE*fm* isolates persistently. In this study, many informative features were identified in the ranges from 3,000 to 4,000 Da, which overlapped with those crucial m/z peaks reported in several previous studies (Lasch et al., 2014; Wei et al., 2014; Wang et al., 2021). Moreover, some informative features were present in the VRE*fm* or VSE*fm* isolates, respectively. The findings of VRE*fm*-specific features in 6,602–6,608 Da has been reported that the peak *m/z* 6,603 is specific for vanB-positive VRE*fm* (Griffin et al., 2012). These VRE*fm*-specific features are worthy of further characterization in further investigations.

Indeed, the VRE*fm* prediction model may detect specific resistant clones instead of the resistant mechanisms. Although the investigation into the resistant mechanism of VRE*fm* is a thoughtful question that we cannot totally figure out in the current stage, we conducted strain typing for the VRE*fm* isolates to illustrate the basic molecular composition. We randomly selected 455 VRE*fm* isolates from blood cultures collected in the institutes over time (2002–2015). In the 455 isolates, only 4 isolates (0.88%) were *vanB, vanA* was predominant (> 99%). Then, we examined the clones by using a multi-locus sequence for the 455 VRE*fm* isolates. The results showed that a total of 24 ST types were identified (Supplementary Figure 2). For testing algorithm on isogenic strains of *E. faecium*, we focused on ST17 as the representative strain and tested ML model performance on discriminating ST17 with *van* gene and ST17 without *van* gene. The ML model attained sensitivity 0.89, specificity 0.8, and AUROC 0.9 in discriminating VSE*fm* ST17 from VRE*fm* ST17. Moreover, the high diverse clone composition for the VRE*fm* isolates has implied that there are some resistance-conferring peptides/proteins in common among various ST types. Given the high diversity of the clones over time, the VRE*fm* prediction model could actually detect the pattern of peaks associated with vancomycin resistance. A total understanding of the underlying mechanism depends on comprehensive identifications of the tens of the informative peaks. Identification of the discriminating peaks would be a tough task that necessaries efforts from the scientific community. In the study, we aimed to develop and validate a novel data preprocessing method that can dig out the implicit information from existing MALDI-TOF spectra for predicting the AST of vancomycin. Meanwhile, the CNN models also provided a list of informative features that are worthy of further molecular investigation to fully understand the underlying mechanism of drug resistance.

There are some limitations to this study. First, the bacterial strains vary according to the environments and locations. Because our data were collected from two tertiary different medical centers in Taiwan, our trained CNN models may not be universally suitable to predict the VRE*fm* strains in other areas or countries. However, the CNN model algorithm is believed to be a powerful method for classifying VRE*fm* strains in clinical applications. Second, our primary goal was to develop and validate a practical and ready-to-use CNN model in clinical practice using whole MALDI-TOF MS spectra. As mentioned earlier we reported some crucial informative peak features for VRE*fm*, however, it is worthy of further confirmation in the identities for these specific peaks corresponding to which peptide products experimentally.

In conclusion, the CNN model was designed to be used in clinical practice. Based on the design, the input is a whole-cell MALDI-TOF MS spectrum that is routinely used for species identification in the clinical microbiology laboratory. Thus, no additional experiment is needed by clinical microbiologists. Once an isolate is identified as *E. faecium*, the raw MALDI-TOF MS spectrum will be transferred to the CNN model directly. Susceptible or resistant to vancomycin will be predicted in seconds and can be provided to clinical physicians.

## Data Availability Statement

The original contributions presented in the study are included in the article/Supplementary Files, further inquiries can be directed to the corresponding author/s.

## Author Contributions

H-YW, J-TH, J-JL, and J-HH conceived the idea and designed the study. T-TH developed the computational algorithms and performed the data analysis. C-RC and H-CC provided assistance in data analysis and interpretation of the results. H-YW and J-HH interpreted the results and wrote the manuscript. All authors contributed to amending the manuscript and have read the submitted version. All authors contributed to the article and approved the submitted version.

## Funding

This work was supported by Chang Gung Memorial Hospital (Linkou) [CMRPG3L0401 (J-JL), CMRPG3L0431 (J-JL), and CMRPG3L1011 (H-YW)] and the Ministry of Science and Technology, Taiwan [111-2320-B-182A-002-MY2 (H-YW)].

## Conflict of Interest

T-TH, H-CC, and J-HH were employed by Taiwan AI Labs. The remaining authors declare that the research was conducted in the absence of any commercial or financial relationships that could be construed as a potential conflict of interest.

## Publisher's Note

All claims expressed in this article are solely those of the authors and do not necessarily represent those of their affiliated organizations, or those of the publisher, the editors and the reviewers. Any product that may be evaluated in this article, or claim that may be made by its manufacturer, is not guaranteed or endorsed by the publisher.
