# Supplementary material for: Efficiently Predicting Vancomycin Resistance of Enterococcus Faecium From MALDI-TOF MS Spectra Using a Deep Learning-Based Approach
Source: Front Microbiol. 2022 Jun 6;13:821233. doi: 10.3389/fmicb.2022.821233 (PMC9231590; doi:10.3389/fmicb.2022.821233)
Supplement: Supplementary file 1 [file Data_Sheet_1.docx]

Efficiently predicting vancomycin resistance of *Enterococcus faecium* from MALDI-TOF MS spectra using a deep learning-based approach

Hsin-Yao Wang^1,2†^, Tsung-Ting Hsieh^3†^, Chia-Ru Chung^4^, Hung-Ching Chang^3^, Jorng-Tzong Horng^1,4,5*^, Jang-Jih Lu^1,6,7*^, Jia-Hsin Huang^3*^

^1^Department of Laboratory Medicine, Chang Gung Memorial Hospital at Linkou, Taoyuan City, Taiwan

^2^Ph.D. Program in Biomedical Engineering, Chang Gung University, Taoyuan City, Taiwan

^3^Taiwan AI Labs, Taipei City 10351, Taiwan

^4^Department of Computer Science and Information Engineering, National Central University, Taoyuan City, Taiwan

^5^Department of Bioinformatics and Medical Engineering, Asia University, Taichung City, Taiwan

^6^School of Medicine, Chang Gung University, Taoyuan City, Taiwan

^7^Department of Medical Biotechnology and Laboratory Science, Chang Gung University, Taoyuan City, Taiwan

^†^The authors contributed equally to this work

*** Correspondence:**

Jorng-Tzong Horng, PhD
[horng@db.csie.ncu.edu.tw](mailto:horng@db.csie.ncu.edu.tw)

Jang-Jih Lu, MD, PhD
[janglu45@gmail.com](mailto:janglu45@gmail.com)

Jia-Hsin Huang, PhD
[jiahsin.huang@ailabs.tw](mailto:jiahsin.huang@ailabs.tw)

**Supplementary Materials**

**Supplementary Table 1**. Primary parameters for the two ML models.

| **ML model** | **RF** | **XGBoost** |
| --- | --- | --- |
| Paprameters | max_depth = 13 | gamma = 0.1 |
|  | n_estimators = 350 | eta = 0.1 |
|  | Bootstrap = True | lambda = 2 |
|  |  | max_depth = 8 |
|  |  | subsample = 0.7 |
|  |  | min_child_weight = 3 |
|  |  | colsample_bytree = 0.7 |
|  |  | num_boost_round = 100 |


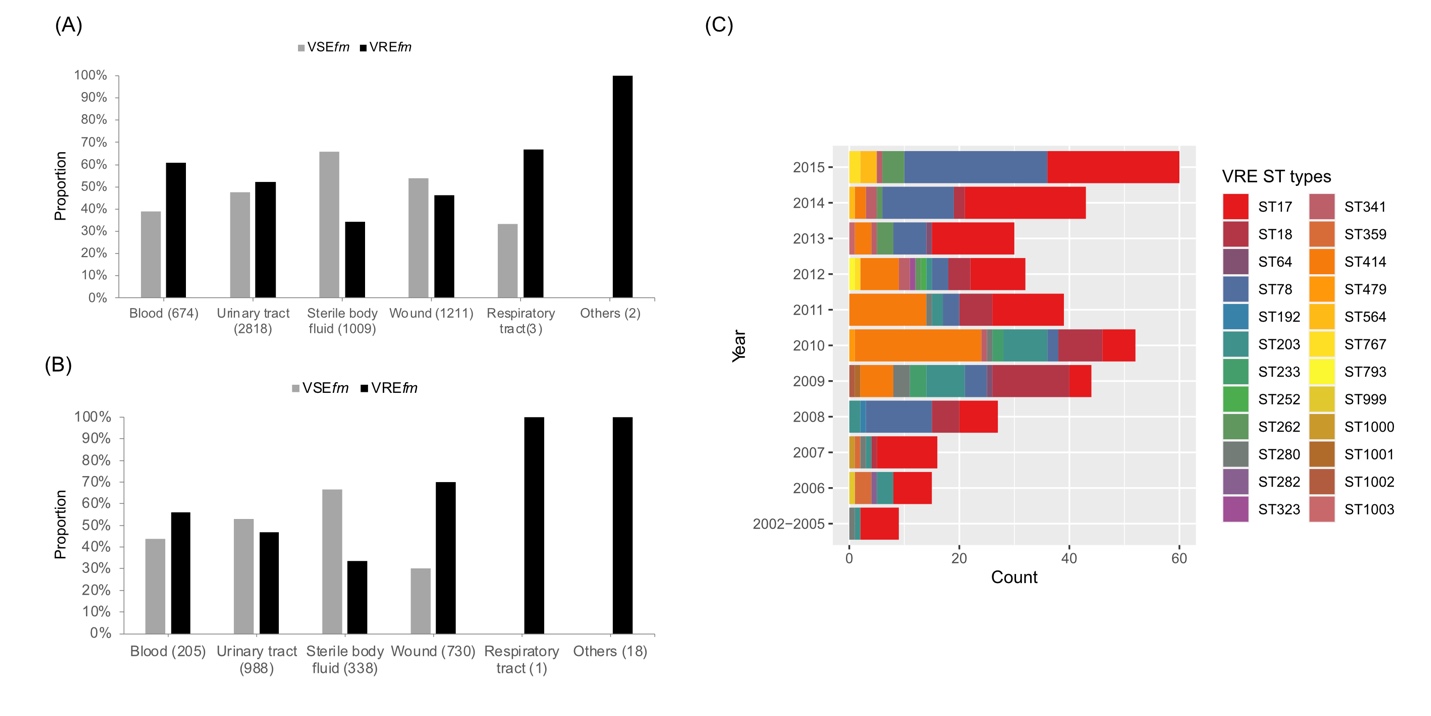


**Supplementary Figure 1.** **Distribution of *Enterococcus faecium*** **bacterial isolates in the clinical samples.** Proportions of the VRE*fm* and VSE*fm* isolates collected from different types of specimens from the CGMH Linkou branch (A) and the CGMH Kaohsiung branch (B). Total sample sizes are shown in the parentheses.


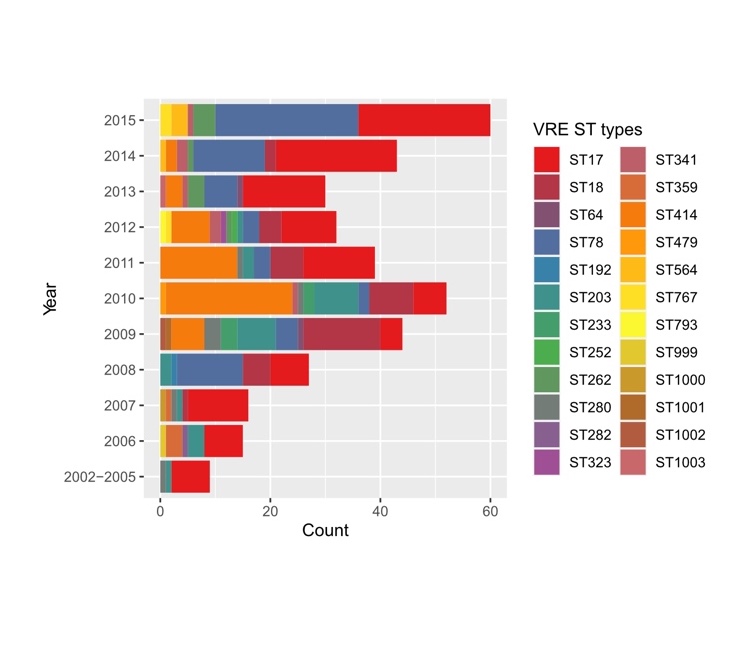


**Supplementary Figure 2. Basic strain composition of VRE*fm* over time in the institutes.** Strain typing (by multi-locus sequence typing) of the 455 VRE*fm* isolates that were selected from blood samples over time. In total, 24 different strains were identified.

**Supplementary Figure 3. MALDI-TOF MS profiles of VRE*fm* isolates and the informative feature peaks.** MALDI-TOF MS spectra of VRE*fm* isolates (ST17 as the example here) are depicted as (A) whole spectrum (*m/z* 2000-20000), (B) *m/z* 2000-3000, (C) *m/z* 3000-4000, (D) *m/z* 4000-5000, (E) *m/z* 5000-6000, and (F) *m/z* 6000-7500. The 30 informative feature peaks are labeled with red rectangles, and the corresponding m/z ranges were annotated as well.
